# Sarcopenia in Urothelial Bladder Carcinoma: A Narrative Review

**DOI:** 10.3390/medicina61071307

**Published:** 2025-07-20

**Authors:** Constantin Radu Vrabie, Andreea Ioana Parosanu, Cornelia Nitipir

**Affiliations:** 1 Faculty of Medicine, Carol Davila University of Medicine and Pharmacy, 050474 Bucharest, Romania; constantin-radu.vrabie@drd.umfcd.ro (C.R.V.); cornelia.nitipir@umfcd.ro (C.N.); 2 Department of Oncology, Brașov Emergency Clinical Hospital, 500326 Brasov, Romania; 3 Department of Oncology, Elias University Emergency Hospital, 011461 Bucharest, Romania; 4 Department of Oncology, Prof. Dr. Agrippa Ionescu Clinical Emergency Hospital, 077015 Balotesti, Romania

**Keywords:** sarcopenia, body composition, bladder urothelial carcinoma, MIBC, NMIBC

## Abstract

*Background and Objectives*: Urothelial bladder carcinoma includes a spectrum of malignant lesions with heterogeneous molecular, biological, and clinical features and a variable risk of progression from non-muscle-invasive bladder cancer (NMIBC) to muscle-invasive disease (MIBC) and ultimately to metastatic urothelial carcinoma (mUC). Sarcopenia, a condition secondary to a catabolic state, is characterized by progressive loss of skeletal muscle mass and function and is highly prevalent across all stages of bladder cancer. This review aims to synthesize current evidence regarding the clinical impact of sarcopenia and its dynamic changes throughout the disease course. *Materials and Methods*: A narrative literature review was conducted using PubMed, Scopus, and Cochrane databases, incorporating the most relevant published sources. Search terms included “bladder carcinoma”, “sarcopenia”, “body composition”, “NMIBC”, and “MIBC”. Case reports and congress abstracts were excluded. *Results*: In NMIBC treated with intravesical Bacillus Calmette–Guérin (BCG), sarcopenia has been shown to have a negative predictive value in some studies. Among patients receiving neoadjuvant chemotherapy (NAC) for MIBC, sarcopenia has been associated with increased toxicity, dose reductions, and treatment delays. In the context of radical surgery, sarcopenia correlates with increased postoperative mortality and a higher rate of severe complications. In mUC, low muscle mass is a negative prognostic factor regardless of treatment type and is associated with chemotherapy-related hematologic toxicity, although it does not appear to predict immune-related adverse events (irAEs). *Conclusions*: Sarcopenia is a highly prevalent and clinically relevant phenotype of urothelial bladder cancer patients, impacting prognosis, treatment response, and chemotherapy toxicity. Incorporating sarcopenia with other relevant components of body composition (BC) and systemic inflammatory markers may facilitate the development of more robust risk scores. Current evidence is primarily limited by the retrospective design of most studies. Future prospective research is needed to clarify the prognostic role of sarcopenia and support its integration into routine clinical decision-making.

## 1. Introduction

The prognosis of bladder carcinoma results from the interplay between tumor biology, treatment response, and the patient’s nutritional and overall health status. Many patients experience unintentional weight loss or a decline in functional role during the disease course. This underscores the importance of BC analysis as a tool to appreciate prognosis and guide therapeutic decisions.

Obesity, which signifies the presence of excessive adipose tissue, is commonly defined using a surrogate measure, body mass index (BMI), which remains one of the most thoroughly investigated components of anthropometry. A BMI greater than 30 kg/m^2^ defines obesity. Excess adiposity is a well-established risk factor for the development of various solid malignancies, particularly breast and colorectal cancers [1].

A comprehensive meta-analysis of 203 studies demonstrated that obesity is associated with decreased overall survival (OS) (hazard ratio (HR) 1.14; 95% confidence interval (CI): 1.09–1.19; *p* < 0.001) and cancer-specific survival (CSS) (HR 1.17; 95% CI: 1.12–1.23; *p* < 0.001). Notably, the data also suggest a paradoxical association between obesity and improved prognosis in certain malignancies, including lung cancer, renal cell carcinoma, and melanoma [1,2].

BMI is a poor predictor of BC, which refers to the distribution of muscle and adipose tissue. Significant interindividual variability exists, even among patients with identical BMI values. Weight loss introduces a dynamic and often unpredictable shift in BC, with the potential for disproportionate reductions in specific tissue compartments [3,4].

According to the diagnostic criteria established by the European Working Group on Sarcopenia in Older People 2 (EWGSOP2), sarcopenia is defined by reduced muscle strength, low muscle quality, and decreased physical performance [5]. In most studies and in clinical practice, particularly in cancer patients, only the quantitative parameter is predominantly assessed.

The skeletal muscle area (SMA) measured on a CT image at the level of the third lumbar vertebra (L3), normalized for height, is referred to as the skeletal muscle index (SMI), which is the most validated metric for assessing sarcopenia [6]. There is considerable heterogeneity in the cut-off values used to define sarcopenia across studies, which complicates interstudy comparisons and clinical application. The most adopted thresholds are those proposed by Prado et al., with SMI < 55 cm^2^/m^2^ for men and SMI < 39 cm^2^/m^2^ for women. Another frequently cited definition is that of Martin et al., which adjusts for BMI, using < 43 cm^2^/m^2^ for men with BMI < 25 kg/m^2^, <53 cm^2^/m^2^ for men with BMI ≥ 25 kg/m^2^, and <43 cm^2^/m^2^ for women regardless of BMI [7,8]. Alternative approaches to muscle mass assessment, including the use of psoas muscle area or volume, have been used in various studies [9]. Nonetheless, the lack of universally accepted cut-off thresholds limits the consistency of these evaluations.

This review aims to synthesize the latest evidence regarding the impact of sarcopenia and its dynamic changes on the clinical trajectory of patients across all spectrums of bladder urothelial carcinoma, NMIBC, MIBC, and mUC. Emphasis will be placed on prognostic and predictive significance, treatment toxicity, and methodological considerations.

## 2. Materials and Methods

**Figure 1:**
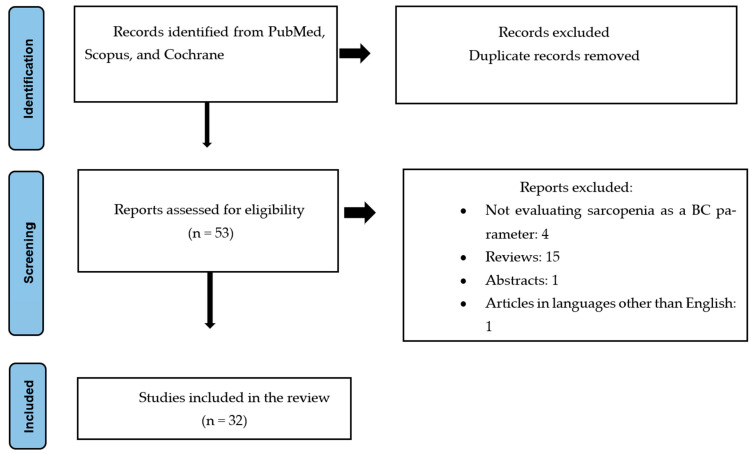
The search strategy and article selection.

A non-systematic literature search of PubMed, Scopus, and Cochrane electronic databases was performed from 1/2010 to 05/2025. A combination of the following keywords was used: bladder carcinoma”, “sarcopenia”, “BC”, “NMIBC”, and “MIBC”. All studies that addressed the potential role of sarcopenia and BC for the prognosis and response to treatment of adult patients with bladder urothelial carcinoma were included. Reviews, congress abstracts, and studies not evaluating sarcopenia as a BC parameter were excluded. 32 studies were included in this review (Figure 1).

## 3. Results

### 3.1. Role of Sarcopenia in NMIBC

NMIBC is a heterogeneous condition with a variable individual risk of local recurrence or progression to MIBC. Transurethral resection of bladder tumor (TURBT) and risk-adjusted adjuvant instillation therapy with BCG or chemotherapy represent the mainstay of treatment. Despite excellent outcomes in most patients, 40% recur within 2 years, and approximately 10% progress to MIBC [10].

Several studies have evaluated the quantity of skeletal muscle and its impact on NMIBC patients treated with intravesical BCG therapy, all of which are retrospective and are summarized in Table 1.

In the retrospective analysis of Alam et al., sarcopenia was highly prevalent (61.1%) but was not associated with recurrence rate, radical cystectomy rate, or treatment-related events [11]. In a separate cohort of 185 patients with high-risk T1 NMIBC, published by Soria et al., 70% were classified as sarcopenic. In this study, sarcopenia and BMI were predictive of disease progression to MIBC (HR 2.72, 95% CI: 1.05–7.02; *p* = 0.04) but were not associated with OS, CSS, or relapse-free survival (RFS) [12].

A retrospective study by Liu et al. investigated the role of preoperative sarcopenia and systemic immune inflammation index (SII) in predicting response to intravesical BCG therapy in intermediate- and high-risk NMIBC patients. Among 183 patients analyzed, sarcopenia and high SII were significantly associated in multivariable analysis with BCG non-response (OR 0.169; 95% CI: 0.064–0.447; *p* < 0.001) and RFS (HR 2.040; 95% CI: 1.250–3.333; *p* < 0.005) [13]. In the study of Huang et al., sarcopenia was a negative predictor for RFS and OS, and it correlated with fewer BCG instillations [14].

### 3.2. Role of Sarcopenia in MIBC Treated with NAC

Neoadjuvant cisplatin-based chemotherapy (NAC) is currently the standard of care for patients with MIBC who are eligible for cisplatin treatment, yielding an 8% absolute survival benefit in this patient population [15]. However, this benefit is accompanied by the risk of toxicity and a decline in overall fitness, which may limit some individuals’ operability window. There is an unmet need for reliable biomarkers to enhance patient selection.

Several recent, but all of them retrospective studies evaluated skeletal muscle quantity and the dynamics of BC changes during NAC and are summarized in Table 2 [16,17,18,19,20,21,22].

NAC induces lean mass loss ranging from 2.6% to 6.4% [16,17,19,20,21]. In the study of Zargar et al., the proportion of psoas muscle volume loss was associated with the need for dose reduction or treatment delays (coefficient B 4.6; 95% CI: 0.05–9.2; *p* = 0.047) [20]. Most of the studies have not identified sarcopenia as an independent predictor of oncological outcomes or treatment response [18,19,20]. Interestingly, in the work of Lyon et al., only post-NAC sarcopenia was found to have negative prognostic value (HR 1.9; 95% CI: 1.02–3.56; *p* = 0.04) concerning CSS [17].

The combination of sarcopenia and obesity was found to be predictive of severe NAC toxicity (*p* = 0.003) in the study of MacDonald et al. [21]. In a retrospective cohort, patients with sarcopenia and normal FMI had a 3-year CSS of 55% compared to 69% in those with sarcopenia and obesity by fat mass index and 88% in those with normal BC parameters (*p* = 0.03) [22].

### 3.3. Impact of Sarcopenia on Surgical and Oncological Outcomes in MIBC

Radical cystectomy alongside lymphadenectomy remains the cornerstone of curative treatment of MIBC, or very high-risk or refractory NMIBC [23]. This extensive urological procedure is associated with a high rate of perioperative mortality, up to 5%, and significant morbidity, with 90-day complication rates ranging from 36.1% to 80.5%, even in tertiary centers [24,25]. While several predictors of postoperative outcomes have been recognized, incorporating them into a comprehensive risk assessment tool is an unfulfilled requirement in modern personalized surgery [26]. Although methods of calculating muscle mass and cut-off values varied widely between studies, sarcopenia was reported in 32.5–68.8% of patients. Most studies identified preoperative sarcopenia as an independent predictor for OS and CSS and are summarized in Table 3 [27,28,29,30,31,32,33,34,35,36,37].

Mayr et al. demonstrated that sarcopenia is a predictor of both 90-day postoperative mortality (OR 2.59; 95% CI 1.13–5.95; *p* = 0.025) and severe complications (Clavien–Dindo grade ≥ 3b-5) (OR 2.84; 95% CI 1.33–6.01; *p* = 0.007) [34]. Using PMI to define sarcopenia, Saitoh-Maeda et al. reported similar findings, with sarcopenic patients experiencing a significantly higher overall complication rate (82.9% vs. 31.8%, *p* < 0.001) and a greater incidence of severe postoperative complications (Clavien–Dindo grade ≥ 3: 19.5% vs. 0%, *p* < 0.001) [27]. Smith et al. found sarcopenia to be predictive of major complications only for the female population (OR 2.2, 95% CI: 1.1–4.6, *p* = 0.02), but it did not show an effect on OS [29]. Psutka et al. showed a trend towards higher 90-day post-radical cystectomy mortality rate in sarcopenic patients (7.8% vs. 1.6%, *p* = 0.07) but did not reach statistical significance. It also demonstrated sarcopenia as a negative prognostic factor for CSS (HR 2.14; *p* = 0.007) and OS (HR 1.63; *p* = 0.007) [30].

In a retrospective cohort, Ha et al. reported that postoperative sarcopenia also has prognostic value. An SMI loss greater than 2.2% was associated with reduced postoperative survival (HR 2.689; 95% CI: 1.007–7.719, *p* = 0.048) [31]. Furthermore, in their multicenter analysis, Mayr et al. identified sarcopenia as an independent negative prognostic factor for both OS (HR 1.42; 95% CI: 1.00–2.02; *p* = 0.048) and CSS (HR 1.42; 95% CI: 1.00–2.02; *p* = 0.048) [37].

### 3.4. Impact of Sarcopenia in Advanced Urothelial Bladder Carcinoma

Cisplatin-based chemotherapy has long been the mainstay of treatment for mUC, with a 5-year OS rate of approximately 15% [38]. The introduction of immunotherapy, either as a second-line treatment or as maintenance therapy following platinum-based chemotherapy, prolongs survival in selected patients [39,40]. More recently, a combination of the anti-PD-1 antibody pembrolizumab and the antibody-drug conjugate enfortumab vedotin has significantly improved survival [41].

Despite advances in systemic therapy, we still do not have reliable biomarkers to tailor the optimal treatment. In this context, BC parameters, including skeletal muscle mass and fat distribution, have emerged as potential prognostic and predictive biomarkers in patients with mUC.

Palliative cytotoxic chemotherapy can exacerbate muscle wasting and myosteatosis. These factors, in turn, increase treatment-related toxicity, particularly hematologic toxicity, leading to treatment delays, dose reductions, and reduced overall treatment intensity [42].

Table 4 summarizes the most relevant studies that evaluate the impact of sarcopenia on various clinical outcomes in patients with advanced bladder carcinoma undergoing systemic therapy [43,44,45,46,47,48,49,50,51,52].

In the study of Yumioka et al., sarcopenia assessed by TPA is a negative prognostic factor regarding OS (HR 2.309; 95% CI: 1.021–5.225, *p* = 0.045) and is a predictor of neutropenia (OR: 3.526, CI 95% 1.128–11.01, *p* = 0.030). In a retrospective cohort, Fukushima et al. found that the recovery of muscle mass after 2 cycles of chemotherapy, defined as ΔSMI > 0, correlates with better OS (HR = 0.21, *p* < 0.001) and PFS (HR = 0.94, *p* = 0.001) [52].

Several other studies have reported the negative prognostic impact of baseline sarcopenic status in patients receiving checkpoint inhibitor therapy in second- and third-line treatment settings [49,50,51]. Only the study of Fukushima et al. reported sarcopenia as a negative predictor of response to anti-PD-1 inhibitor pembrolizumab [49].

To better stratify patients treated with ICI, Martini et al. developed a BC risk score by selecting the most statistically relevant variables, integrating SMI, attenuated muscle mean (SM), and visceral fat index (VFI). Patients with intermediate or high-risk scores demonstrated significantly poorer outcomes compared to those with low-risk scores, both in terms of overall survival (OS: 2.7 months vs. 8.9 months vs. not reached; *p* = 0.0011) and progression-free survival (PFS: 1.9 months vs. 3.4 months vs. 10.4 months; *p* = 0.0014) [50].

## 4. Discussions

Sarcopenia is a common characteristic among patients with NMIBC undergoing intravesical BCG therapy, with reported prevalence rates ranging widely from 30% to 70% [11,12,13,14]. One possible explanation for this variability is the lack of consensus regarding the definition of sarcopenia, the cut-off values used, and significant median age differences between cohorts. Consequently, the interpretation of findings related to oncologic outcomes should be approached with caution. Among the selected studies, only the analysis by Huang et al. identified sarcopenia as a predictor for OS. In the same study, sarcopenic patients received fewer BCG instillations, which may have, in turn, impacted prognosis [14]. The study by Soria et al. incorporated sarcopenia to enhance the predictive value of the EORTC risk score for progression to MIBC, potentially aiding in the more rigorous selection of patients [12,53].

It has been recognized since 2004 that chemotherapy induces sarcopenia, which, in turn, contributes to a vicious cycle: sarcopenia increases chemotherapy-related toxicity, reduces treatment compliance, and ultimately compromises therapeutic efficacy, thereby impairing prognosis [54,55].

Cisplatin, the backbone of NAC for urothelial carcinoma, induces muscle wasting through several molecular mechanisms, including disruption of the ubiquitin–proteasome system, alterations in intracellular calcium homeostasis, activation of the PI3K/AKT pathway, and mitochondrial lesions [56]. However, neither the percentage decrease in muscle mass nor the presence of sarcopenia before NAC is prognostic for survival or predictive of pathological response to treatment [16,17,18,19,20,21].

The only BC parameter with prognostic value was identified in the study by Lyon et al., which demonstrated that post-NAC sarcopenia is correlated with CSM [17]. Moreover, NAC has been shown to increase the risk of cisplatin-induced nephrotoxicity in sarcopenic patients in one study, a finding that may be explained by pharmacokinetic factors, as well as the overestimation of renal function when assessed using creatinine clearance in sarcopenic patients [18,57]. In the prospective PLATISMA study, conducted in the context of head and neck carcinoma treated with cisplatin, low muscle mass was associated with higher levels of protein-bound cisplatin, which may contribute to increased toxicity [58].

Sarcopenia is a well-recognized predictor of mortality and morbidity in the surgical field [59]. In oncologic surgery, it is strongly associated with poor outcomes, particularly in the gastrointestinal domain [60]. In the context of radical cystectomy for MIBC, sarcopenia has been consistently correlated with decreased CSS and OS, as well as with the incidence and severity of postoperative complications [27,28,30,31,32,33,34,35,36,37].

In contrast, the study by Smith et al. did not identify a significant association between survival and muscle mass, which was assessed using total psoas area (TPA) [29]. The validity of these findings is limited by the short follow-up period of only 16 months, as well as by the use of a non-validated method for evaluating sarcopenia. In the study by Ha et al., a loss of more than 2.2% in muscle mass was a better predictor of OS than the presence of sarcopenia per se, highlighting the importance of dynamic monitoring of BC [31].

Nonetheless, the conclusions drawn from these studies should be interpreted with caution due to their retrospective nature, heterogeneity in muscle mass assessment methods, variability in cut-off values, and inconsistent reporting of postoperative complications.

Despite these limitations, sarcopenia should not be regarded merely as a marker of frailty. Rather, it represents a quantifiable, independent risk factor for adverse surgical and oncological outcomes in patients undergoing radical cystectomy.

Among patients with advanced bladder carcinoma undergoing first-line platinum-based chemotherapy, sarcopenia has been consistently identified as an adverse prognostic factor, despite considerable heterogeneity in study populations and variability in the methodologies employed for its assessment [43,44,45,46,48,52].

In the studies by Yumioka et al. and Gao et al., sarcopenia was identified as a predictor of chemotherapy-induced neutropenia and leukopenia, suggesting that patients with sarcopenia may benefit from primary prophylaxis against febrile neutropenia [45,47]. In contrast, Fukushima et al. found no significant association between sarcopenia and immune-related adverse events (irAEs), reporting an incidence of 21% in sarcopenic patients compared to 33% in non-sarcopenic patients (*p* = 0.48) [49]. This discrepancy highlights the possibility that low muscle mass may differentially influence toxicity profiles depending on the treatment modality. One proposed mechanism is that reduced lean body mass in sarcopenic individuals decreases the volume of distribution for cytotoxic agents, potentially leading to drug overexposure. Additionally, diminished muscle mass may result in deceptively low serum creatinine levels, leading to an overestimation of renal function and inappropriate dosing of renally excreted chemotherapeutic agents [61].

Due to small sample sizes, retrospective study designs, and the absence of a standardized definition of sarcopenia, the current body of evidence remains limited, underscoring the need for prospective validation.

Sarcopenia is a distinct prognostic factor in oncology, comprising two main components: primary sarcopenia, which is related to patient-specific factors such as age, performance status, and nutritional condition, and secondary sarcopenia, which is driven by intrinsic features of the neoplastic process, including systemic inflammation and a catabolic metabolic profile or treatment-related [62]. Post-therapeutic sarcopenic status has been established as a prognostic factor across various studies, regardless of the clinical setting [17,31,43].

In patients with urothelial carcinoma of the bladder, secondary sarcopenia often overlaps with primary sarcopenia. This convergence likely contributes to the high prognostic relevance of sarcopenia, as consistently reported in the literature [63].

## 5. Conclusions

Sarcopenia is a highly prevalent and clinically meaningful trait of urothelial bladder cancer patients, impacting prognosis, treatment response, and chemotherapy toxicity. Although sarcopenia demonstrates its strongest prognostic value in advanced-stage disease, irrespective of treatment modality, its significance in early-stage bladder cancer remains less well defined.

In the context of radical cystectomy, skeletal muscle mass should be part of a pre-treatment risk stratification model, ideally incorporating objective evaluations of physiological age, physical performance, nutritional status, lean body mass, and frailty.

Incorporating sarcopenia with other relevant components of BC and systemic inflammatory markers may facilitate the development of more robust risk scores, thereby improving patient stratification and guiding treatment selection.

Future prospective research is needed to clarify the prognostic role of sarcopenia and support its integration into routine clinical decision-making.

## Figures and Tables

**Table 1:** Key studies investigating the impact of sarcopenia in NMIBC.

Authors (Year)	Patients No.	Age (Years)	Stage	Assessment	Sarcopenia Cut-Off		Sarcopenic (%)	Study Results
					Men (cm^2^/m^2^) Women (cm^2^/m^2^)			
Alam et al. (2023) [11].	90	75.2	Ta T1	CT–L3	<55	<39	61%	RR*, RC*, TRE*; no association with SMI
Soria et al. (2023) [12].	185	71	T1	CT-L3	<55	<39	70%	SMI predictive of DP* (HR: 2.72, 95% CI: 1.05–7.02; *p* < 0.04), but no association with OS and CSS
Liu et al. (2022) [13].	183	62.3	Ta T1	CT-L3	<43 for BMI < 25 kg/m^2^ <53 for BMI > 25 kg/m^2^	<43	39.9%	Sarcopenia predictive for RFS (*p* = 0.005) and BCG non-response (*p* < 0.0001)
Huang et al. (2023) [14].	269	66	Ta T1	CT-L3	<45.4	<34.4	29.7%	Sarcopenia predicts poorer RFS and OS (*p* = 0.030 and *p* = 0.033)

DP—disease progression, RR—recurrence rate, RC—radical cystectomy, SMI—skeletal muscle index, TRE—treatment-related events, RFS—relapse-free survival, HR—hazard ratio, CSS—cancer-specific survival.

**Table 2:** Key studies investigating the impact of sarcopenia and muscle loss in patients undergoing NAC on oncological outcomes.

Authors (Year)	Patient No.	Follow-Up	Method of Assessment	Sarcopenia Cut Off		Sarcopenia Prevalence	Muscle Loss	Study Results
				Men (cm^2^/m^2^) Women (cm^2^/m^2^)				
Stangl-Kremser et al. (2018) [16].	30	NR	CT-L3, SMI	<55 cm^2^/m^2^	<39 cm^2^/m^2^	53.3%	3%	No association of SMI (*p* = 0.78 and *p* = 0.59), sarcopenia (*p* = 0.65 and *p* = 0.16) or SMI kinetics (*p* = 0.54 and *p* = 0.77) with CR or PR
Lyon et al. (2019) [17].	183	36 months	CT-L3, SMI	<55 cm^2^/m^2^	<39 cm^2^/m^2^	55%	8.4%	Post-NAC sarcopenia correlates with CSM (HR, 1.90; 95% CI: 1.02–3.56; *p* = 0.04). No BC measurements associated with OS or treatment response
Regnier et al. (2021) [18].	82	26 months	CT-L3, SMI	<50 cm^2^/m^2^	35 cm^2^/m^2^	57.3%	NR	Sarcopenia is not associated with RFS, CSS, OS, or PR (*p* = 0.066) but predicts cisplatin renal toxicity (OR 3.01; 95% CI: 1.13–8.05; *p* = 0.02) and early postoperative complications (≤90 days) (OR 4.08; 95% CI: 1.06–15.6; *p* = 0.04)
Rimar et al. (2018) [19].	26	NR	CT-L3, SMI	<55 cm^2^/m^2^	<38.5 cm^2^/m^2^	69.2%	6.4%	Sarcopenia is not associated with treatment response (*p* = 0.66)
Zargar et al. (2017) [20].	60	NR	CT, PMV	NR	NR	NR	4.9%	PMV loss was not associated with RFS (HR 0.98; 95% CI: 0.91–1.05; *p* = 0.55), CSS (HR 1.003; 95% CI: 0.92–1.09; *p* = 0.95) or OS (HR 1.01, 95% CI: 0.95–1.08; *p* = 0.74)
MacDonald et al. (2024) [21]	70	NR	CT-L3, SMI	<55 cm^2^/m^2^	<39 cm^2^/m^2^	54%	3.4%	NAC decreases SMI. SO predictive of severe NAC toxicity (≥grade 3) (OR 8.37; 95% CT: 2.06–34.6; *p* = 0.003)
Diamantopoulos et al. (2020) [22].	143	2.7 years	CT-L3, SMI	<55 cm^2^/m^2^	<39 cm^2^/m^2^	60%	NR	Sarcopenia + normal FMI 3-year CSS (55%) vs. sarcopenia + FMI-obesity (79%), normal SMI with FMI-obesity (69%), and normal BC (88%, *p* = 0.03)

CT—computed tomography; SMI—skeletal muscle index; CR—clinical response; PR—pathological response; NAC—neoadjuvant chemotherapy; HR—hazard ratio; CSS—cancer-specific survival; RFS—relapse-free survival; OS—overall survival; SO—sarcopenic obesity; FMI—fat mass index; PMV—psoas muscle volume; NR—not reported; CSM—cancer-specific mortality; BC—body composition.

**Table 3:** Key studies investigating the impact of sarcopenia on surgical and oncological outcomes for MIBC.

Authors (Year)	Patients No.	Age (Median)	Follow-Up	Assessment Method	Sarcopenia Cut-Off (cm^2^/m^2^)	Prevalence	Oncological Outcomes	Surgical Outcomes
Psutka et al. (2014) [30].	205	71	6.7 years	CT-L3, SMI	Men: <55, Women: <39	68.8% before RC	Sarcopenia independently predicted CSS (HR 2.14; *p* = 0.007), OS (HR 1.63, *p* = 0.007) and all-cause mortality (HR, 1.93; *p* = 0.004)	A trend towards higher 90-day mortality rate, 7.8% in sarcopenic pts vs. 1.6% in nonsarcopenic pts (*p* = 0.07)
Mayr et al. (2018) [34].	327	70	90 days	CT-L3, SMI	Men < 43 for BMI < 25 kg/m^2^, and <53 for BMI ≥ 25 kg/m^2^ and Women: <41	33% before RC	NR	Sarcopenia independently predicted 90 dM (OR 2.59; 95% CI 1.13–5.95; *p* = 0.025) and a higher rate of major complications; Clavien–Dindo ≥ 3b-5 (OR 2.84 (1.33–6.01) *p* = 0.007)
Smith et al. (2017) [29].	200	66.6	1.4 years	CT-L3, TPA	Men: <653, Women: <523	50% before RC	No association between OS and sarcopenia (*p* = 0.36)	Sarcopenia in women is an independent predictor of major complications (OR 2.2, 95% CI 1.1–4.6, *p* = 0.02)
Saitoh-Maeda et al. (2017) [27].	78	NR	NR	CT-umbilical level, PMI	Low PMI < 400, High PMI ≥ 400	NR	High PMI and better OS vs. low PMI (*p* = 0.023)	Low PMI patients’ complication rate was 82.9% vs. 31.8% (*p* < 0.001), and low PMI predicts high-grade surgical complications (Clavien grade ≥ 3, 19.5% vs. 0%, *p* < 0.001)
Ying et al. (2021) [28].	292	74	3.8 years	CT-AI calculated SMV	NR	NR	Low MV independently predicts OS (HR 1.62 (1.07–2.44, *p* = 0.022)	MV below the median was not associated with high-grade complications (OR 1.30, 95% CI 0.73–2.32; *p* = 0.380)
Engelmann et al. (2023) [35].	657	70	40 months	CT-L3, SMI	Men < 43 for BMI < 25 kg/m^2^, and <53 for BMI ≥ 25 kg/m^2^ and Women: <41	51.8% before RC	Sarcopenia independent prognostic factor for OS (HR = 1.59, CI: 1.27–2.00; *p* < 0.01); CSS (HR 1.87, CI: 1.40–2.51, *p* < 0.01)	NR
Ha et al. (2019) [31].	81	NR	46.2 months	CT-L3, SMI	Men < 43 for BMI < 25 kg/m^2^, and <53 for BMI ≥ 25 kg/m^2^ and Women: <41	32.5% sarcopenic before RC and 50.0% sarcopenic after RC	Patients with post-RC sarcopenia had higher all-cause mortality rates than those without sarcopenia (*p* = 0.012). SMI loss of ≥2.2 cm^2^/m^2^ after RC (HR: 2.689, 95% CI: 1.007–7.719, *p* = 0.048) was found to be an independent predictor of OS	NR
Yamashita et al. (2021) [32].	123	74	39 months	CT-L3 SMI	Men: <40.8; Women: <34.9	39% before RC	Sarcopenia independently predicts poor OS (*p* = 0.02) and CSS (*p* < 0.01)	NR
Hirasawa et al. (2016) [33].	136	68.6	46.7 months	CT-L3 SMI	Men: <55 Women: <39	47.8% before RC	Sarcopenia independently predicts poor CSS (HR = 2.3; *p* = 0.015)	NR
Mao et al. (2020) [36].	200	66.0	NR	CT-L3 TPI	Women: <385 mm^2^/m^2^ or TPI <545 mm^2^/m^2^ for Male Patients.	33.5% before RC	Sarcopenia has prognostic value regarding OS (*p* = 0.016) and DFS (*p* = 0.023).	NR
Mayr et al. [37].	500	72	22 months	CT-L3 SMI	Men < 43 for BMI < 25 kg/m^2^, and <53 for BMI ≥ 25 kg/m^2^ and Woman: <41	37.8% before RC	Sarcopenia predicted OS (HR 1.42; 95% CI, 1.00–2.02; *p* = 0.048) and CSS (HR 1.42; 95% CI, 1.00–2.02; *p* = 0.048)	NR

CT—computed tomography; SMI—skeletal muscle index; RC—radical cystectomy; SMV—skeletal muscle volume; TPA—total psoas area; TPI—total psoas index; PMI—psoas muscle mass index; L3—third lumbar vertebra; HR—hazard ratio; CSS—cancer-specific survival; RFS—relapse-free survival; OS—overall survival; dM—day mortality; DFS—disease-free survival; MV—muscle volume; NR—not reported; AI—artificial intelligence.

**Table 4:** Key studies investigating the impact of sarcopenia on prognosis and treatment toxicities in advanced urothelial bladder carcinoma.

Authors (Year)	N	Age (Median)	Follow-Up Months	Assessment Method	Sarcopenia Cut-Off (cm^2^/m^2^)	Prevalence	Treatment Regimen	Treatment Toxicity	Meaningful Findings
Gao et al. (2024) [45].	112	56	NR	CT-L3 PMI	Men: 4.5 cm^2^/m^2^ Women: 3.3 cm^2^/m^2^	38.4% before treatment	Tislelizumab + GC	Leukopenia (OR 2.969, 95% CI 1.028–8.575, *p* = 0.044)	No difference in ORR (*p* = 0.606) or DCR (*p* = 0.988).
Taguchi et al. (2015) [46].	64	68	NR	CT-L3, SMI, TPA	Men: <55, Women: <39	NR	MVAC, GC, GCa, DIP	NR	Sarcopenia predicts poor CSS (HR, 2.07; 95% CI: 1.01–4.67, *p* = 0.045)
Abe et al. (2017) [44].	87	73	15.4	CT-L3, SMI, TPA, PSMI	Men: <55, Women: <39	89% before treatment	GC, GCa	NR	Sarcopenia stratified by obesity (BMI > 25) independently predicted OS (HR 3.102, 95% CI: 1.149–8.374, *p* = 0.026)
Fukushima et al. (2014) [48].	88	68	13	CT-L3 SMI	Men: <43 for BMI < 25 kg/m^2^, and <53 for BMI ≥ 25 kg/m^2^ and Women: <41	60% before treatment	NR	NR	OS: 11 months for sarcopenic and 31 months for non-sarcopenic patients (*p* < 0.001), and an independent predictor of shorter OS (HR 3.36; *p* < 0.001)
Fukushima et al. (2020) [49].	30	74	6	CT-L3 SMI	Men < 43 for BMI < 25 kg/m^2^, and <53 for BMI ≥ 25 kg/m^2^ and Women: <41	68%	Pembrolizumab 2L	irAEs not related to sarcopenia	ORR: 67% for non-sarcopenic vs. 21% for sarcopenic pts (*p* = 0.019). Trend towards better OS for non-sarcopenic: median, not reached vs. 7 months (*p* = 0.086). PFS: sarcopenic pts (median, 3 vs. 15 months, *p* = 0.038 for non-sarcopenic pts)
Borelli et al. (2023) [43].	97	NR	17.3	CT-L3, SMI, AI software	Men: <55, Women: <39	53.6%	GC, GCa	NR	Baseline sarcopenia did not predict OS, but post-CHT sarcopenic status, both by SMI-L3 coefficient and cut-off, was found to independently predict clinical benefits (OR: 0.93, 95% CI: 0.88–0.98, *p* = 0.006 and OR: 2.31, 95% CI: 1.15–5.78, *p* = 0.038)
Yumioka et al. (2019) [47].	80	71.6	NR	CT-L3, TPA	Men: 4.57 cm^2^/m^2^ Women: 3.35 cm^2^/m^2^	48.7%	GC, GCa	Sarcopenia predictive of neutropenia (OR: 3.526, CI 95% 1.128–11.01, *p* = 0.030)	No impact on treatment response (*p* = 0.406). Sarcopenia was an independent predictive factor of OS in multivariate analysis (HR 2.309; 95% CI: 1.021–5.225, *p* = 0.045)
Martini et al. (2021) [50].	70	69.5	20.1	CT-L3, SMI	NR	NR	ICI 2L and 3L	NR	High- and intermediate-risk BC patients had worse prognosis OS (2.7 months versus 8.9 months versus not reached; *p* = 0.0011) and PFS (1.9 months versus 3.4 months versus 10.4 months; *p* = 0.0014) compared with low-risk patients
Shimizu et al. (2020) [51].	29	NR	7	CT-L3, PMI	Men: 6.36 cm^2^/m^2^ Women: 3.92 cm^2^/m^2^	56%	2L or 3L pembrolizumab	NR	Sarcopenia independently predicts poor OS (HR 1.99, 95% CI: 0.50–7.49, *p*= 0.040) and PFS (HR 2.79, 95% CI 1.14–7.32, *p* = 0.030)
Fukushima et al. (2017) [52].	72	68	13	CT-L3, SMI	Men < 43 for BMI < 25 kg/m^2^, and <53 for BMI ≥ 25 kg/m^2^ and Women: <41	67% ΔSMI median −5.2 (−22.1 to 23.5)	MVAC, GC, GCA	PSR not associated with toxicity, *p* = 0.071	ΔSMI predicted PFS (HR = 0.94, *p* = 0.001) and OS (HR = 0.93, *p* = 0.001). PSR was an independent predictor for both PFS (HR = 0.24, *p* < 0.001) and OS (HR = 0.21, *p* < 0.001)

GC: gemcitabine + cisplatin; GCa: gemcitabine + carboplatin; MVAC: methotrexate + vinblastine + doxorubicin + cisplatin; irAEs: immune-related adverse events; ORR: objective response rate; DCR: disease control rate; PFS: progression-free survival; OS: overall survival; CSS: cancer-specific survival; TPA: total psoas area; ΔSMI: [(post-therapeutic SMI − pre-therapeutic SMI)/pre-therapeutic SMI] × 100; PSR: post-therapeutic muscle mass recovery = ΔSMI > 0; pts: patients; NR: not reported; 2L/3L: second line/third line.

## Data Availability

No new data were created or analysed in this study. Data sharing is not applicable to this article.

## Abbreviations

The following abbreviations are used in this manuscript:

NMIBC	non-muscle-invasive bladder cancer
mUC	metastatic urothelial carcinoma
MIBC	muscle-invasive disease
BCG	Bacillus Calmette–Guérin
NAC	neoadjuvant chemotherapy
irAEs	immune-related adverse events
BMI	body mass index
BC	body composition
HR	hazard ratio
SMA	skeletal muscle area
SMI	skeletal muscle index
TURBT	Transurethral resection of bladder tumor
OS	overall survival
RFS	relapse-free survival
CSS	cancer-specific survival
FMI	fat mass index
VFI	visceral fat index

## References

[1] Petrelli F., Cortellini A., Indini A., Tomasello G., Ghidini M., Nigro O., Salati M., Dottorini L., Iaculli A., Varricchio A. (2021). Association of Obesity With Survival Outcomes in Patients With Cancer: A Systematic Review and Meta-analysis. JAMA Netw. Open.

[2] Pati S., Irfan W., Jameel A., Ahmed S., Shahid R.K. (2023). Obesity and Cancer: A Current Overview of Epidemiology, Pathogenesis, Outcomes, and Management. Cancers.

[3] Fearon K., Strasser F., Anker S.D., Bosaeus I., Bruera E., Fainsinger R.L., Jatoi A., Loprinzi C., MacDonald N., Mantovani G. (2011). Definition and classification of cancer cachexia: An international consensus. Lancet Oncol..

[4] Baracos V.E., Arribas L. (2018). Sarcopenic obesity: Hidden muscle wasting and its impact for survival and complications of cancer therapy. Ann. Oncol..

[5] Cruz-Jentoft A.J., Bahat G., Bauer J., Boirie Y., Bruyère O., Cederholm T., Cooper C., Landi F., Rolland Y., Sayer A.A. (2019). Sarcopenia: Revised European consensus on definition and diagnosis. Age Ageing.

[6] Anjanappa M., Corden M., Green A., Roberts D., Hoskin P., McWilliam A., Choudhury A. (2020). Sarcopenia in cancer: Risking more than muscle loss. Tech. Innov. Patient Support Radiat. Oncol..

[7] Martin L., Birdsell L., MacDonald N., Reiman T., Clandinin M.T., McCargar L.J., Murphy R., Ghosh S., Sawyer M.B., Baracos V.E. (2013). Cancer Cachexia in the Age of Obesity: Skeletal Muscle Depletion Is a Powerful Prognostic Factor, Independent of Body Mass Index. J. Clin. Oncol..

[8] Prado C.M., Lieffers J.R., McCargar L.J., Reiman T., Sawyer M.B., Martin L., Baracos V.E. (2008). Prevalence and clinical implications of sarcopenic obesity in patients with solid tumours of the respiratory and gastrointestinal tracts: A population-based study. Lancet Oncol..

[9] Hartmann V., Engelmann S.U., Pickl C., Haas M., Kälble S., Goßler C., Eckl C., Hofmann A., Pichler R., Burger M. (2024). Impact of sarcopenia and fat distribution on outcomes in penile cancer. Sci. Rep..

[10] Matulewicz R.S., Steinberg G.D. (2020). Non–muscle-invasive Bladder Cancer: Overview and Contemporary Treatment Landscape of Neoadjuvant Chemoablative Therapies. Rev. Urol..

[11] Alam S.M., Larson M., Srinivasan P., Genz N., Fleer R., Sardiu M., Thompson J., Lee E., Hamilton-Reeves J., Wulff-Burchfield E. (2023). Evaluation of sarcopenia in patients receiving intravesical Bacillus Calmette-Guérin for non-muscle invasive bladder cancer. Urol. Oncol. Semin. Orig. Investig..

[12] Soria F., D’Andrea D., Barale M., Gust K.M., Pisano F., Mazzoli S., De Bellis M., Rosazza M., Livoti S., Dutto D. (2023). Sarcopenia Predicts Disease Progression in Patients with T1 High-grade Non–muscle-invasive Bladder Cancer Treated with Adjuvant Intravesical Bacillus Calmette-Guérin: Implications for Decision-making?. Eur. Urol. Open Sci..

[13] Liu P., Chen S., Gao X., Liang H., Sun D., Shi B., Zhang Q., Guo H. (2022). Preoperative sarcopenia and systemic immune-inflammation index can predict response to intravesical Bacillus Calmette-Guerin instillation in patients with non-muscle invasive bladder cancer. Front. Immunol..

[14] Huang L.K., Lin Y.C., Chuang H.H., Chuang C.K., Pang S.T., Wu C.T., Chang Y.-H., Yu K.-J., Lin P.-H., Kan H.-C. (2023). Body composition as a predictor of oncological outcome in patients with non-muscle-invasive bladder cancer receiving intravesical instillation after transurethral resection of bladder tumor. Front. Oncol..

[15] Yin M., Joshi M., Meijer R.P., Glantz M., Holder S., Harvey H.A., Kaag M., van de Putte E.E.F., Horenblas S., Drabick J.J. (2016). Neoadjuvant Chemotherapy for Muscle-Invasive Bladder Cancer: A Systematic Review and Two-Step Meta-Analysis. Oncologist.

[16] Stangl-Kremser, Mari A., D’Andrea D., Kimura S., Resch I., Shariat S.F., Klatte T. (2018). Sarcopenia as a Predictive Factor for Response to Upfront Cisplatin-Based Chemotherapy in Patients with Muscle-Invasive Urothelial Bladder Cancer. Urol. Int..

[17] Lyon T.D., Frank I., Takahashi N., Boorjian S.A., Moynagh M.R., Shah P.H., Tarrell R.F., Cheville J.C., Viers B.R., Tollefson M.K. (2019). Sarcopenia and Response to Neoadjuvant Chemotherapy for Muscle-Invasive Bladder Cancer. Clin. Genitourin. Cancer.

[18] Regnier P., De Luca V., Brunelle S., Sfumato P., Walz J., Rybikowski S., Maubon T., Branger N., Fakhfakh S., Durand M. (2021). Impact of sarcopenia status of muscle-invasive bladder cancer patients on kidney function after neoadjuvant chemotherapy. Minerva Urol. Nephrol..

[19] Rimar K.J., Glaser A.P., Kundu S., Schaeffer E.M., Meeks J., Psutka S.P. (2018). Changes in Lean Muscle Mass Associated with Neoadjuvant Platinum-Based Chemotherapy in Patients with Muscle Invasive Bladder Cancer. Bladder Cancer.

[20] Zargar H., Almassi N., Kovac E., Ercole C., Remer E., Rini B., Stephenson A., Garcia J.A., Grivas P. (2017). Change in Psoas Muscle Volume as a Predictor of Outcomes in Patients Treated with Chemotherapy and Radical Cystectomy for Muscle-Invasive Bladder Cancer. Bladder Cancer.

[21] MacDonald L., Rendon R.A., Thana M., Wood L., MacFarlane R., Bell D., Duplisea J., Mason R. (2024). An in-depth analysis on the effects of body composition in patients receiving neoadjuvant chemotherapy for urothelial cell carcinoma. Can. Urol. Assoc. J..

[22] Diamantopoulos L.N., Ngo S., Maldonado R., O’Malley R.B., Laidlaw G., Fintelmann F.J., Holt S.K., Gore J.L., Schade G.R., Lin D.W. (2020). Associations between baseline body composition and cancer-specific mortality following neoadjuvant chemotherapy and radical cystectomy for bladder cancer. J. Clin. Oncol..

[23] Aminoltejari K., Black P.C. (2020). Radical cystectomy: A review of techniques, developments and controversies. Transl. Androl. Urol..

[24] Sobhani S., Ghoreifi A., Douglawi A., Ahmadi H., Miranda G., Cai J., Aron M., Schuckman A., Desai M., Gill I. (2023). Perioperative mortality for radical cystectomy in the modern Era: Experience from a tertiary referral center. Int. Braz. J. Urol..

[25] Maibom S.L., Joensen U.N., Poulsen A.M., Kehlet H., Brasso K., Røder M.A. (2021). Short-term morbidity and mortality following radical cystectomy: A systematic review. BMJ Open.

[26] Razdan S., Sljivich M., Pfail J., Wiklund P.K., Sfakianos J.P., Waingankar N. (2021). Predicting morbidity and mortality after radical cystectomy using risk calculators: A comprehensive review of the literature. Urol. Oncol. Semin. Orig. Investig..

[27] Saitoh-Maeda Y., Kawahara T., Miyoshi Y., Tsutsumi S., Takamoto D., Shimokihara K., Hayashi Y., Mochizuki T., Ohtaka M., Nakamura M. (2017). A low psoas muscle volume correlates with a longer hospitalization after radical cystectomy. BMC Urol..

[28] Ying T., Borrelli P., Edenbrandt L., Enqvist O., Kaboteh R., Trägårdh E., Ulén J., Hayashi Y., Kjölhede H. (2021). Automated artificial intelligence-based analysis of skeletal muscle volume predicts overall survival after cystectomy for urinary bladder cancer. Eur. Radiol. Exp..

[29] Smith A.B., Deal A.M., Yu H., Boyd B., Matthews J., Wallen E.M., Pruthi R.S., Woods M.E., Muss H., Nielsen M.E. (2014). Sarcopenia as a Predictor of Complications and Survival Following Radical Cystectomy. J. Urol..

[30] Psutka S.P., Carrasco A., Schmit G.D., Moynagh M.R., Boorjian S.A., Frank I., Stewart S.B., Thapa P., Tarrell R.F., Cheville J.C. (2014). Sarcopenia in patients with bladder cancer undergoing radical cystectomy: Impact on cancer-specific and all-cause mortality. Cancer.

[31] Ha Y.S., Kim S.W., Kwon T.G., Chung S.K., Yoo E.S. (2019). Decrease in skeletal muscle index one year after radical cystectomy as a prognostic indicator in patients with urothelial bladder cancer. Int. Braz. J. Urol..

[32] Yamashita S., Iguchi T., Koike H., Wakamiya T., Kikkawa K., Kohjimoto Y., Hara I. (2021). Impact of preoperative sarcopenia and myosteatosis on prognosis after radical cystectomy in patients with bladder cancer. Int. J. Urol..

[33] Hirasawa Y., Nakashima J., Yunaiyama D., Sugihara T., Gondo T., Nakagami Y., Horiguchi Y., Ohno Y., Namiki K., Ohori M. (2016). Sarcopenia as a Novel Preoperative Prognostic Predictor for Survival in Patients with Bladder Cancer Undergoing Radical Cystectomy. Ann. Surg. Oncol..

[34] Mayr R., Fritsche H.M., Zeman F., Reiffen M., Siebertz L., Niessen C., Pycha A., van Rhijn B.W.G., Burger M., Gierth M. (2018). Sarcopenia predicts 90-day mortality and postoperative complications after radical cystectomy for bladder cancer. World J. Urol..

[35] Engelmann S.U., Pickl C., Haas M., Kaelble S., Hartmann V., Firsching M., Lehmann L., Gužvić M., van Rhijn B.W.G., Breyer J. (2023). Body Composition of Patients Undergoing Radical Cystectomy for Bladder Cancer: Sarcopenia, Low Psoas Muscle Index, and Myosteatosis Are Independent Risk Factors for Mortality. Cancers.

[36] Mao W., Ma B., Wang K., Wu J., Xu B., Geng J., Zhang H., Chen M. (2020). Sarcopenia predicts prognosis of bladder cancer patients after radical cystectomy: A study based on the Chinese population. Clin. Transl. Med..

[37] Mayr R., Gierth M., Zeman F., Reiffen M., Seeger P., Wezel F., Pycha A., Comploj E., Bonatti M., Ritter M. (2018). Sarcopenia as a comorbidity-independent predictor of survival following radical cystectomy for bladder cancer. J. Cachexia Sarcopenia Muscle.

[38] Von Der Maase H., Sengelov L., Roberts J.T., Ricci S., Dogliotti L., Oliver T., Moore M.J., Zimmermann A., Arning M. (2005). Long-Term Survival Results of a Randomized Trial Comparing Gemcitabine Plus Cisplatin, With Methotrexate, Vinblastine, Doxorubicin, Plus Cisplatin in Patients With Bladder Cancer. J. Clin. Oncol..

[39] Powles T., Park S.H., Voog E., Caserta C., Valderrama B.P., Gurney H., Kalofonos H., Radulović S., Demey W., Ullén A. (2020). Avelumab Maintenance Therapy for Advanced or Metastatic Urothelial Carcinoma. N. Engl. J. Med..

[40] Bellmunt J., De Wit R., Vaughn D.J., Fradet Y., Lee J.L., Fong L., Vogelzang N.J., Climent M.A., Petrylak D.P., Choueiri T.K. (2017). Pembrolizumab as Second-Line Therapy for Advanced Urothelial Carcinoma. N. Engl. J. Med..

[41] Powles T., Valderrama B.P., Gupta S., Bedke J., Kikuchi E., Hoffman-Censits J., Iyer G., Vulsteke C., Park S.H., Shin S.J. (2024). Enfortumab Vedotin and Pembrolizumab in Untreated Advanced Urothelial Cancer. N. Engl. J. Med..

[42] Choi M.H., Yoon S.B. (2022). Sarcopenia in pancreatic cancer: Effect on patient outcomes. World J. Gastrointest. Oncol..

[43] Borrelli A., Pecoraro M., Del Giudice F., Cristofani L., Messina E., Dehghanpour A., Landini N., Roberto M., Perotti S., Muscaritoli M. (2023). Standardization of Body Composition Status in Patients with Advanced Urothelial Tumors: The Role of a CT-Based AI-Powered Software for the Assessment of Sarcopenia and Patient Outcome Correlation. Cancers.

[44] Abe H., Takei K., Uematsu T., Tokura Y., Suzuki I., Sakamoto K., Nishihara D., Yamaguchi Y., Mizuno T., Nukui A. (2018). Significance of sarcopenia as a prognostic factor for metastatic urothelial carcinoma patients treated with systemic chemotherapy. Int. J. Clin. Oncol..

[45] Gao Z., Pang Y., Qin X., Li G., Wang Z., Zhang L., Wang J., Qi N., Li H. (2024). Sarcopenia is associated with leukopenia in urothelial carcinoma patients who receive tislelizumab combined with gemcitabine and cisplatin therapy. Int. J. Clin. Oncol..

[46] Taguchi S., Akamatsu N., Nakagawa T., Gonoi W., Kanatani A., Miyazaki H., Fujimura T., Fukuhara H., Kume H., Homma Y. (2016). Sarcopenia Evaluated Using the Skeletal Muscle Index Is a Significant Prognostic Factor for Metastatic Urothelial Carcinoma. Clin. Genitourin. Cancer.

[47] Yumioka T., Honda M., Nishikawa R., Teraoka S., Kimura Y., Iwamoto H., Morizane S., Hikita K., Takenaka A. (2020). Sarcopenia as a significant predictive factor of neutropenia and overall survival in urothelial carcinoma patients underwent gemcitabine and cisplatin or carboplatin. Int. J. Clin. Oncol..

[48] Fukushima H., Yokoyama M., Nakanishi Y., Tobisu K., Koga F. (2015). Sarcopenia as a Prognostic Biomarker of Advanced Urothelial Carcinoma. PLoS ONE.

[49] Fukushima H., Fukuda S., Moriyama S., Uehara S., Yasuda Y., Tanaka H., Yoshida S., Yokoyama M., Matsuoka Y., Fujii Y. (2020). Impact of sarcopenia on the efficacy of pembrolizumab in patients with advanced urothelial carcinoma: A preliminary report. Anticancer Drugs.

[50] Martini D.J., Shabto J.M., Goyal S., Liu Y., Olsen T.A., Evans S.T., Magod B.L., Ravindranathan D., Brown J.T., Yantorni L. (2021). Body Composition as an Independent Predictive and Prognostic Biomarker in Advanced Urothelial Carcinoma Patients Treated with Immune Checkpoint Inhibitors. Oncologist.

[51] Shimizu T., Miyake M., Hori S., Ichikawa K., Omori C., Iemura Y., Owari T., Itami Y., Nakai Y., Anai S. (2020). Clinical Impact of Sarcopenia and Inflammatory/Nutritional Markers in Patients with Unresectable Metastatic Urothelial Carcinoma Treated with Pembrolizumab. Diagnostics.

[52] Fukushima H., Kataoka M., Nakanishi Y., Sakamoto K., Takemura K., Suzuki H., Ito M., Tobisu K.I., Fujii Y., Koga F. (2018). Posttherapeutic skeletal muscle mass recovery predicts favorable prognosis in patients with advanced urothelial carcinoma receiving first-line platinum-based chemotherapy. Urol. Oncol. Semin. Orig. Investig..

[53] Cambier S., Sylvester R.J., Collette L., Gontero P., Brausi M.A., Van Andel G., Kirkels W.J., Silva F.C., Oosterlinck W., Prescott S. (2016). EORTC Nomograms and Risk Groups for Predicting Recurrence, Progression, and Disease-specific and Overall Survival in Non–Muscle-invasive Stage Ta–T1 Urothelial Bladder Cancer Patients Treated with 1–3 Years of Maintenance Bacillus Calmette-Guérin. Eur. Urol..

[54] Freedman R.J., Aziz N., Albanes D., Hartman T., Danforth D., Hill S., Sebring N., Reynolds J.C., Yanovski J.A. (2004). Weight and Body Composition Changes during and after Adjuvant Chemotherapy in Women with Breast Cancer. J. Clin. Endocrinol. Metab..

[55] Bozzetti F. (2017). Forcing the vicious circle: Sarcopenia increases toxicity, decreases response to chemotherapy and worsens with chemotherapy. Ann. Oncol..

[56] Conte E., Bresciani E., Rizzi L., Cappellari O., De Luca A., Torsello A., Liantonio A. (2020). Cisplatin-Induced Skeletal Muscle Dysfunction: Mechanisms and Counteracting Therapeutic Strategies. Int. J. Mol. Sci..

[57] Okamura M., Konishi M., Butler J., Kalantar-Zadeh K., Von Haehling S., Anker S.D. (2023). Kidney function in cachexia and sarcopenia: Facts and numbers. J. Cachexia Sarcopenia Muscle.

[58] Chargi N., Molenaar-Kuijsten L., Huiskamp L.F.J., Devriese L.A., De Bree R., Huitema A.D.R. (2022). The association of cisplatin pharmacokinetics and skeletal muscle mass in patients with head and neck cancer: The prospective PLATISMA study. Eur. J. Cancer.

[59] Knoedler S., Schliermann R., Knoedler L., Wu M., Hansen F.J., Matar D.Y., Obed D., Vervoort D., Haug V., Hundeshagen G. (2023). Impact of sarcopenia on outcomes in surgical patients:A systematic review and meta-analysis. Int. J. Surg..

[60] Wang H., Yang R., Xu J., Fang K., Abdelrahim M., Chang L. (2021). Sarcopenia as a predictor of postoperative risk of complications, mortality and length of stay following gastrointestinal oncological surgery. Ann. R. Coll. Surg. Engl..

[61] Hilmi M., Jouinot A., Burns R., Pigneur F., Mounier R., Gondin J., Neuzillet C., Goldwasser F. (2019). Body composition and sarcopenia: The next-generation of personalized oncology and pharmacology?. Pharmacol. Ther..

[62] Fukushima H., Takemura K., Suzuki H., Koga F. (2018). Impact of Sarcopenia as a Prognostic Biomarker of Bladder Cancer. Int. J. Mol. Sci..

[63] Wiedmer P., Jung T., Castro J.P., Pomatto L.C.D., Sun P.Y., Davies K.J.A., Grune T. (2021). Sarcopenia—Molecular mechanisms and open questions. Ageing Res. Rev..

